# Functionalisable Epoxy-rich Electrospun Fibres Based on Renewable Terpene for Multi-Purpose Applications

**DOI:** 10.3390/polym13111804

**Published:** 2021-05-30

**Authors:** Ulisse Montanari, Davide Cocchi, Tommaso Maria Brugo, Antonino Pollicino, Vincenzo Taresco, Maria Romero Fernandez, Jonathan C. Moore, Domenico Sagnelli, Francesca Paradisi, Andrea Zucchelli, Steven M. Howdle, Chiara Gualandi

**Affiliations:** 1 School of Chemistry, University Park University of Nottingham, Nottingham NG7 2RD, UK; Ulisse.Montanari1@nottingham.ac.uk (U.M.); Vincenzo.Taresco@nottingham.ac.uk (V.T.); Maria.Romerofernandez@nottingham.ac.uk (M.R.F.); J.Moore@nottingham.ac.uk (J.C.M.); domenico.sagnelli@isasi.cnr.it (D.S.); francesca.paradisi@dcb.unibe.ch (F.P.); 2 Department of Chemistry “Giacomo Ciamician” and INSTM UdR of Bologna, University of Bologna, Via Selmi, 2, 40126 Bologna, Italy; 3 Department of Industrial Engineering (DIN), Università di Bologna, Viale del Risorgimento 2, 40123 Bologna, Italy; davide.cocchi5@unibo.it (D.C.); tommasomaria.brugo@unibo.it (T.M.B.); a.zucchelli@unibo.it (A.Z.); 4 Interdepartmental Center for Industrial Research on Advanced Applications in Mechanical Engineering and Materials Technology, CIRI-MAM, University of Bologna, Viale Risorgimento, 2, 40136 Bologna, Italy; 5 D.I.C.A.R., Dipartimento di Ingegneria Civile e Architettura University of Catania, Viale Andrea Doria 6, 95125 Catania, Italy; apollicino@unict.it; 6 Department of Chemistry and Biochemistry, University of Bern, Freiestrasse 3, 3012 Bern, Switzerland

**Keywords:** bio-based polymer, electrospinning, surface functionalisation, epoxy group, carbon fibre reinforced composites, enzyme immobilisation

## Abstract

New bio-based polymers capable of either outperforming fossil-based alternatives or possessing new properties and functionalities are of relevant interest in the framework of the circular economy. In this work, a novel bio-based polycarvone acrylate di-epoxide (PCADE) was used as an additive in a one-step straightforward electrospinning process to endow the fibres with functionalisable epoxy groups at their surface. To demonstrate the feasibility of the approach, poly(vinylidene fluoride) (PVDF) fibres loaded with different amounts of PCADE were prepared. A thorough characterisation by TGA, DSC, DMTA and XPS showed that the two polymers are immiscible and that PCADE preferentially segregates at the fibre surface, thus developing a very simple one-step approach to the preparation of ready-to-use surface functionalisable fibres. We demonstrated this by exploiting the epoxy groups at the PVDF fibre surface in two very different applications, namely in epoxy-based carbon fibre reinforced composites and membranes for ω-transaminase enzyme immobilisation for heterogeneous catalysis.

## 1. Introduction

The valorisation of waste streams from agricultural and industrial sources into a bio-based supply chain for the production of valuable materials is a central topic of the circular economy [1,2]. A great deal of effort is currently undertaken by the bio-based industries and research centres to find and exploit new waste sources as well as to implement biorefinery technologies and pathways towards cost-competitive products with low environmental impact. Competitive bio-based polymers must demonstrate cost and/or performance improvements with respect to fossil-based alternatives. In particular, bio-based plastics can become particularly relevant if their properties outperform those of conventional plastics in advanced applications [3,4,5].

As biomass feedstocks, terpenes show great promise because they are affordable and available at scale for polymerisation. As an example, polymers synthesised using limonene as feedstock are among the top 20 most innovative bio-based products showing the greatest promise for commercial deployment within the next 5–10 years [6]. Terpene derivatives can be used to synthesise several types of homopolymers and copolymers with a range of properties [7], including polyesters [8], polyamides [9], polyurethanes [10] and polycarbonates [11]. Besides the plastic industry, terpenes are of relevant interest as protective agents [12], green solvents [13] and catalysts [14].

In this framework, we have recently demonstrated the synthesis of a novel polycarvone acrylate di-epoxide (PCADE) as a less toxic and bio-based alternative to poly(glycidyl methacrylate) [15]. The most intriguing aspect of this polymer is the presence of two epoxy groups per repeating unit, potentially exploitable for chemical modifications, thus making PCADE a platform for the synthesis of multi-functional materials. Additionally, it could be envisaged that the polymer may be employed as a surface coater and promoter of surface chemical modifications to achieve specific functionalities. Surface functionalisation is a relevant topic on a technologically point of view since it can improve substrate performance with minimal effects on bulk properties. Materials with high surface-to-volume ratio, a typical example being electrospun submicrometric fibres, could achieve enormous benefit from the use of PCADE as a promoter of surface functionalisation. Briefly, electrospinning uses electrostatic forces to stretch a viscoelastic polymer solution and produces fibres with diameters in the range of few tenths of nanometers to a few micrometers. Fibres are deposited on a collector as non-wovens, having mesh porosity typically higher than 80% and pore diameters that can vary from a few to tens of micrometers [16,17]. Electrospun fibres can be exploited for a range of applications, including filtration, sensors, catalytic systems, in energy harvesting/conversion/storage, in structural composites and in biomedical applications. Moreover, electrospinning not only is a consolidate technology on a lab-scale level but the scaling up has been realised in the past decade by several companies.

The possibility to easily control and modify the surface properties of electrospun fibres is crucial for many applications and several approaches can be envisaged [18], such as plasma treatments [19,20,21], physical vapour deposition [22,23] and covalent grafting of polymers and other active species [24,25,26,27,28]. Covalent modification is the preferred approach to achieve specific and efficient fibre surface functionalisation, albeit often it requires multiple steps, including the generation of anchor functionalities on the fibre surface, followed by the attachment of linking agents, if needed, and eventually the introduction of molecules providing the desired functionality. To avoid multiple steps and elaborate synthetic pathways, the spontaneous surface segregation of a functional additive at the fibre surface during electrospinning process can be exploited [29,30].

**Figure 1:**
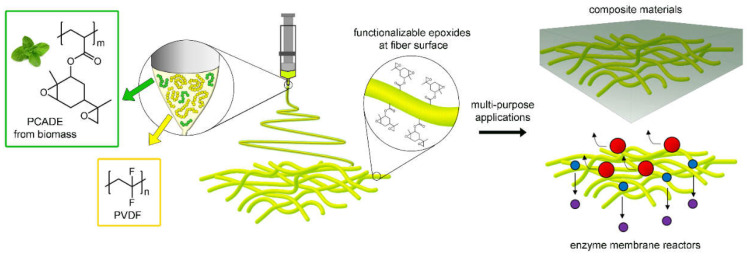
PCADE and PVDF were blended in a common solution and electrospun to produce fibres with an epoxy-rich surface. Different PVDF/PCADE fibres were tested in epoxy CFRP and for enzyme immobilisation.

In the present work PCADE was used as surface modifier of the fibres and electrospun in blend with poly(vinylidene fluoride) (PVDF). A low molecular weight PCADE was intentionally employed to promote its migration towards the jet surface during electrospinning to obtain an epoxy-enriched fibre surface. The two polymers were blended in different ratios, in order to tailor the fibre toughness and the epoxy content at fibre surface. The resulting non-wovens were thoroughly characterised to determine their morphology, phase separation, bulk and surface chemical composition and mechanical properties. PVDF/PCADE electrospun fibres were then tested in two different applications where the surface chemistry is of paramount importance—as second fillers in Carbon Fibre Reinforced Polymers (CFRP) and to form membranes for enzyme immobilisation for heterogeneous catalysis (Figure 1).

## 2. Materials and Methods

### 2.1. Fibre Production and Characterisation

#### 2.1.1. Materials

Poly(carvone acrylate di-epoxide) (PCADE, *M*_n_ = 2800 g mol^−1^, PDI = 1.5) was synthesised as previously described [15] and reported in the Supplementary Materials (Figures S1 and S2). Polyvinylidene fluoride (PVDF, 6008, Solef) was kindly provided by Solvay Specialty Polymers. Acetone (Ac, ACS reagent, 99.5%) and dimethylformamide (DMF, ACS reagent, 99.8%) were purchased from Sigma-Aldrich and used without further purification.

#### 2.1.2. Electrospinning

Nanofibrous mats were fabricated using a laboratory electrospinning machine (Spinbow Lab Unit, Spinbow S.r.1., Bologna, Italy), equipped with a linear sliding spinneret carrying two syringes ejecting the same polymer solution at a rate of 1.2 mL h^−1^ and connected to two stainless-steel blunt-ended needles (inner diameter= 0.51 mm) with PTFE tubes. A drum collector (diameter = 150 mm; length = 500 mm) rotating at 50 rpm and positioned 23 cm away from the needles was used as collector. PVDF mats were prepared from a 18% (*w*/*v*) solution of PVDF dissolved in Ac:DMF=60:40 (*v*/*v*), by applying a voltage of 17 kV. PVDF/PCADE 85/15 (*w*/*w*) and PVDF/PCADE 70/30 (*w*/*w*) were prepared from 18% (*w*/*v*) polymer solutions in Ac:DMF = 70:30 (*v*/*v*), by applying a voltage of 25 and 26 kV, respectively. In a typical preparation, 1.53 g of PVDF and 0.27 g of PCADE were dissolved in 10 mL to prepare PVDF/PCADE 85/15, whereas 1.26 g of PVDF and 0.54 of PCADE were dissolved in 10 mL to prepare PVDF/PCADE 70/30. Electrospinning was performed at room temperature (RT) and relative humidity 30–40%. The electrospinning process was carried out until the thickness of the mat was about 40 μm.

#### 2.1.3. Characterisation Techniques

Scanning electron microscopy (SEM) was performed on a Zeiss LEO 1530 FE-SEM, operated at 5 kV. Samples were sputter-coated with gold prior to SEM observations. and observed with an In-lens SE detector. The distribution of fibre diameters was determined through the measurement of about 100 fibres and the results are given as the average diameter ± standard deviation (SD). The one-way ANOVA was used to test the statistical significance of the difference between the mean values (*p* < 0.001).

XPS spectra were recorded with a VG Microtech spectrometer with a CLAMII analyser. The X-ray source (Mg Kα1,2, 1253.6 eV) worked at 100 kV and 10 mA at a pressure < 2 × 10^−8^ Torr. Pass energy: 100 eV for wide scans, 50 eV for narrow scans. The spectra were recorded with taking-off angles of 45°. All data analyses (linear background subtraction and peak integration) were accomplished using PeakFit software (v.4 by SPSS). Binding energies were referenced to the C-H level at 285.0 eV.

Thermogravimetric analyses (TGA) were carried out using a TA instruments Q500 thermogravimetric analyser. Analyses were performed from RT to 700 °C, at a heating rate of 10 °C min^−1^, under air flow.

Thermal transitions were measured by means of a TA Instruments differential scanning calorimeter, DSC Q100, equipped with a refrigerated cooling system (RCS). Samples were subjected to a first heating scan at 10 °C min^−1^ from −50 °C up to 120°C, a controlled cooling at 10°C min^−1^ up to −50°C and a second heating scan at 10 °C min^−1^.

Dynamic mechanical thermal analysis (DMTA) was carried out on electrospun strips (gauge length about 10 mm) by means of a TA Instruments dynamic mechanical, analyser DMA Q800, under a tensile configuration. The specimens were tested within a heating ramp from −90 °C to 100 °C at 3 °C min^−1^, adopting a frequency of 1 Hz under displacement control.

Stress−strain measurements of the mats were performed on an Instron 4465 tensile testing machine equipped with a 100 N load cell. Samples 5 mm wide with a gauge length of 30 mm were pre-clamped on a paper frame following the procedure described in Maccaferri et al. [31]. The tensile test was performed at 10 mm min^−1^ and for each sample six replicate specimens were tested for each type of mat. The stress (σ) was normalised to the real material cross section according to the following equation:

(1) $$\sigma =\rho \;\frac{F}{m}\;L$$

where *ρ* is the material bulk density (approximated to PVDF density for all types of mat), *m* is the specimen’s mass, *L* is specimen’s initial length and *F* is the force. Elastic modulus (E), maximum stress (σ_max_), maximum deformation (ε_max_) and absorbed strain energy (U) of the electrospun samples were provided as mean ± standard deviation.

### 2.2. Carbon Fibre Composites

#### 2.2.1. Materials

Twill 200 gsm carbon-epoxy prepreg (GG204T-IMP503Z), supplied by Angeloni S.r.l., was chosen as the base material. The epoxy resin, according to the supplier datasheet, is based on diglycidyl ether bisphenol A and has a glass transition temperature of 120 °C after curing.

#### 2.2.2. Epoxy Resin−Electrospun Nanofibres Composites

The uncured epoxy was prepared at RT by mixing the pre-polymer and the curing agent at a ratio 100/30 (*w*/*w*) (according to manufacturer’s instructions) and degassed under vacuum. To produce epoxy−nanofibre composites a rectangular electrospun membrane was placed on a PTFE film and the uncured epoxy mixture was gently poured onto it to get a complete impregnation. The excess of uncured resin was eliminated by using filter paper. The impregnated mat was then heated to 90 °C at a rate of 10 °C h^−1^, followed by an isotherm at 90 °C for 1 h. Samples were fractured in liquid nitrogen for SEM characterisation.

#### 2.2.3. CFRP Nano-Interleaved Composite Laminate

Four types of laminates, with dimensions of 120 × 170 mm^2^, were manufactured by stacking 14 plies of CFRP prepreg: (i) an unmodified laminate (FRP-Virgin), (ii) a PVDF nano-interleaved laminate (FRP-PVDF), (iii) a PVDF/PCADE 85/15 nano-interleaved laminate (FRP-85/15) and (iv) a PVDF/PCADE 70/30 nano-interleaved laminate (FRP-70/30). A PTFE film (30 μm thick) was inserted in the ply book mid-plane, in order to trigger the crack. For the nano-interleaved laminates, nanofibrous mats were integrated in the mid-layer, following the procedure described in Brugo et al. [32]. Laminates were cured in an autoclave with a two-stage cycle—a first isotherm at 70 °C for 30 min and a second one at 135 °C for 120 min, with a heating and cooling rate of 2.5 °C min^−1^. Vacuum bag and autoclave pressure were 900 mbar and 6 bar, respectively (Figure S3).

#### 2.2.4. Characterisation Techniques

Mode-I fracture tests were carried out on a servo-hydraulic testing machine, Instron 8033, equipped with a 2 kN load cell, under displacement control condition at a constant speed of 1.5 mm min^−1^. Double cantilever beam (DCB) samples were prepared according to the ASTM D5528 standard [33]: 140 mm long, 20 mm wide, with an initial crack of 45 mm and two aluminum blocks glued on the tip for clamping.

The energy release rate for mode-I (*G_I_*) testing was obtained by the beam theory method (BTM), described in Equation (2):

(2) $$G_{I}=\frac{3P\delta }{2Ba}$$

where *P* is the load, *δ* is the crack opening displacement, *B* is the specimen width and a is the crack length. Three DCB specimens for each kind of sample, extracted from each of the four laminate types, were tested and results were provided as mean ± standard deviation. During each test, load and displacement were recorded and the crack propagation was visually controlled by means of a high-resolution camera focusing on a ruler fixed on the side of the specimen.

### 2.3. Enzyme Immobilisation

#### 2.3.1. Materials

Iminodiacetic acid (IDA), (S)-(–)-α-methylbenzylamine, sodium pyruvate, pyridoxal 5′-phosphate (PLP), monobasic potassium phosphate, dibasic potassium phosphate, sodium borate, sodium chloride, dimethyl sulfoxide (DMSO) and cobalt (II) chloride were purchased from Sigma-Aldrich.

#### 2.3.2. Immobilisation of HeWT on Metal-Derivatised PVDF/PCADE 70/30 Membranes

ω-transaminase from *Halomonas elongata* (HeWT) was expressed and purified as previously described [34]. HeWT was covalently immobilised on PVDF/PCADE 70/30 membranes partially derivatised with Co^2+^ groups, following a previously reported two-step immobilisation strategy [35]. Briefly, epoxy groups of PVDF/PCADE 70/30 fibres were partially modified with iminodiacetic acid (IDA) by adding 500 mL g_membrane_^−1^ solution of 0.1 M sodium borate 2 M IDA 50 mM potassium phosphate pH 8.5, and incubating under gentle shaking for 2 h and 102 h at RT. The resulting modified membranes, 2hIDA and 102hIDA membranes, were washed with H_2_O and added to 5 mL g_membrane_^−1^ solution of 1M NaCl 5 mg mL^−1^ CoCl_2_ 50 mM potassium phosphate pH 6 to coordinate carboxylic acids groups of IDA with Co^2+^. The mixtures were incubated under gentle shaking for 3 h at RT, and resulting partially Co^2+^-derivatised membranes, Co^2+^-2hIDA- and Co^2+^-102hIDA membranes, were washed with H_2_O. A solution of HeWT in 50 mM potassium phosphate pH 8 was added to Co^2+^-2hIDA- and Co^2+^-102hIDA membranes at a ratio of 10 mg_HeWT_ g_membrane_^−1^ and the immobilisation mixtures were incubated under gentle shaking for 18 h at RT. Samples from the liquid phase were taken at different time points, and ω-transaminase activity was measured. After immobilisation, imm-HeWT membranes (imm-HeWT-Co^2+^-2hIDA and imm-HeWT-Co^2+^-102hIDA membranes) were washed with H_2_O and used to assess the recovered activity. In addition, imm-HeWT membranes were incubated in water at 90 °C for 1 h. A sample of the liquid phase was taken and analysed by SDS-page electrophoresis.

#### 2.3.3. ω-Transaminase Activity Assay and Protein Determination

Enzymatic activity was determined following the production of acetophenone using a standard enzymatic assay as reported by Cerioli et al. [34]. Concentration of the purified protein was determined spectrophotometrically as described by Cerioli et al. [34]. The immobilised activity, U g_membrane_^−1^, is defined as the difference between initial offered activity per gram of membrane and remaining activity in the liquid phase per gram of membrane at immobilisation end point. The immobilisation yield (%) is defined as the ratio between immobilised activity, and initial offered activity per gram of membrane.

#### 2.3.4. Recovered Activity Assay after Immobilisation on Membranes

The activity of the immobilised HeWT was determined by weighing an appropriate amount of imm-HeWT membranes (imm-HeWT-Co^2+^-2hIDA- and imm-HeWT-Co^2+^-102hIDA membranes) followed by the addition of 400 mL g_membrane_^-1^ reaction mixture (50 mM potassium phosphate buffer pH 8; 2.5 mM (S)-(–)-α-methylbenzylamine; 2.5 mM pyruvate; 0.25% DMSO; and 0.1 mM pyridoxal 5′-phosphate (PLP)). The immobilised enzyme reaction mixture was gently shaken for 10 min at 25 °C, and the absorbance at 245 nm was recorded every 2 min as single readings using UV-96-well plates. The recovered activity in U g_membrane_^−1^ is defined as µmol of acetophenone formed per minute per gram of imm-HeWT membrane. The percentage of recovered activity (%) is defined as the ratio between recovered activity in U g_membrane_^−1^ and immobilised activity in U g_membrane_^−1^.

## 3. Results

### 3.1. PVDF/PCADE Electrospun Samples

PCADE can be readily synthesised via free radical polymerisation of carvone acrylate di-epoxide, achieving a reasonable control of molecular weight between 2000 Da and 32,000 Da, with the addition of dodecane thiol as transfer agent and different monomer concentration (see Supplementary Materials for details) [15]. In a first attempt, PCADE with the highest molecular weight (*M*_n_ = 32,000 g mol^−1^, Ð = 2.1) was electrospun to produce single-component fibres. The electrospinning process was successful (Figure S4), meaning that in the selected solvent mixture the critical concentration for entanglements was achieved, leading to beaded fibres that turned into continuous fibres upon the increase of solution concentration [36,37]. However, pure PCADE fibres were discontinuous and extremely fragile due to the polymer’s poor mechanical properties. To improve fibre mechanical properties, a second polymer was thus used in combination with PCADE. PVDF was chosen as the main constituent of the electrospun fibres due to its high mechanical strength and chemical, thermal and electrical stabilities [38]. This specialty polymer is widely used in high-tech applications: membranes for water treatment, gas separation, membrane distillation and lithium-ion batteries [39,40] and sensors, transducers, energy harvesting devices and actuators because of its ferroelectric and piezoelectric properties [41,42]. PVDF is a rather inert and chemically stable fossil-based polymer that we intend to modify with the addition of the bio-based PCADE to confer to the final product a partial bio-based content and as well as new chemical groups for further functionalisation. For the preparation of PVDF/PCADE fibres, we intentionally employed a low molecular weight PCADE (*M*_n_ = 2800 g mol^−1^, Ð = 1.5) to exploit the tendency of low-molecular-weight compounds to migrate towards the jet surface during electrospinning and thus to obtain epoxy-enriched fibre surface [29,30,43,44,45].

**Figure 2:**
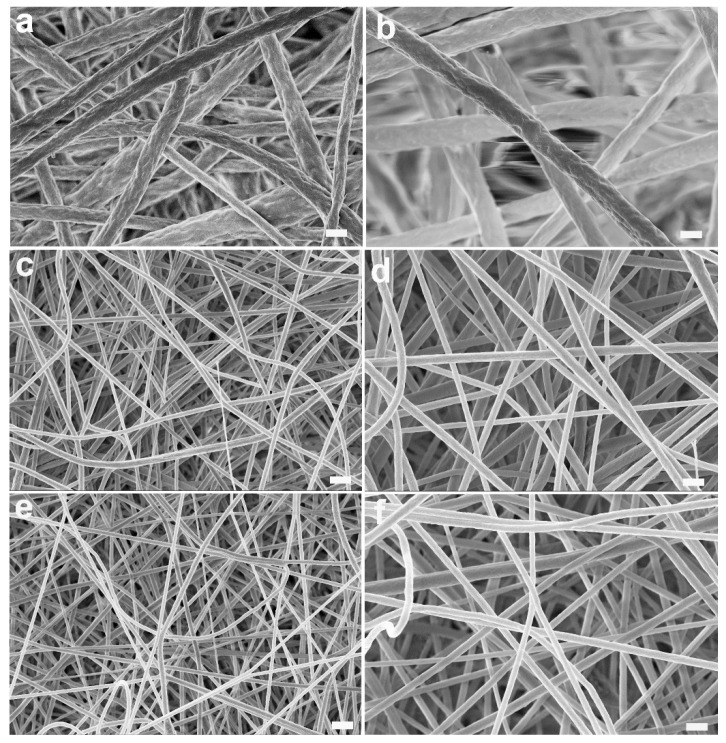
Representative SEM images of electrospun samples: PVDF (**a**,**b**), PVDF/PCADE 85/15 (**c**,**d**) and PVDF/PCADE 70/30 (**e**,**f**). Scale bar: 2 µm (**a**,**c**,**e**) and 1 µm (**b**,**d**,**f**).

PVDF and PCADE were blended in different ratios to tailor fibre toughness and epoxy content at the fibre surface. The resulting mats were characterised to determine their morphology, thermal and mechanical properties, and bulk and surface chemical composition. Three kinds of electrospun samples were produced, one of pure PVDF, to be used as a reference, and two PVDF/PCADE blends in 85/15 and 70/30 ratio (*w*/*w*). Continuous fibres were obtained in each case with diameters of 720 ± 180 nm (PVDF), 400 ± 150 nm (PVDF/PCADE 85/15) and 300 ± 80 nm (PVDF/PCADE 70/30) (Figure 2 and Figure S5). It is evident that fibre diameter significantly decreases with the addition of PCADE (*p* < 0.001 PVDF vs. PVDF/PCADE 85/15) and further decreases when the concentration of PCADE reaches the 30 wt% (*p* < 0.001 PVDF/PCADE 85/15 vs. PVDF/PCADE 70/30). This is determined by the reduced solution viscosity occurring when the high molecular weight PVDF is partially replaced with the low molecular weight PCADE. The decrease of fibre diameter in the presence of PCADE is desirable to increase the surface to volume ratio and maximise the exploitation of epoxide groups at the fibre surface.

**Figure 3:**
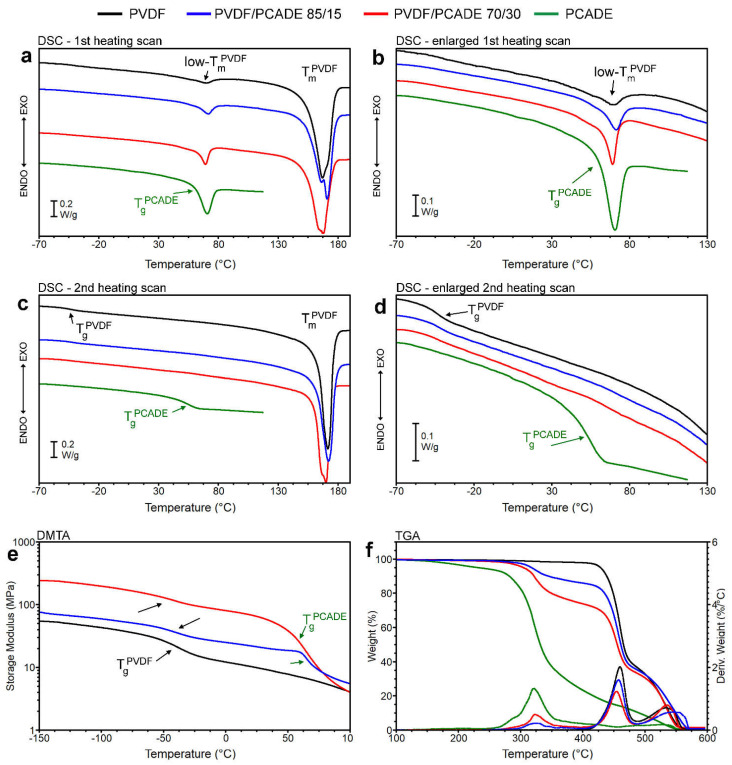
Thermal analysis of electrospun mats. (**a**,**b**) DSC first heating scans; (**c**,**d**) DSC second heating scans after quench; (**e**) storage modulus as a function of temperature determined by DMTA analysis; (**f**) weight loss and its corresponding derivative as a function of temperature determined by TGA analysis. PVDF = black, PVDF/PCADE 85/15 = blue, PVDF/PCADE 70/30 = red and PCADE powder =green. Thermal transitions ascribable to the PVDF phase are indicated with black arrows, whereas those of PCADE with green arrows.

DSC analysis was performed to gain information on the miscibility of PVDF and PCADE in the electrospun fibres. Figure 3a reports the first heating scans and Figure 3b is the corresponding enlargement of the glass transition region; Figure 3c,d, similarly, report the second heating scans after quench. DSC of PVDF fibres clearly shows that this polymer is highly semicrystalline, with a first heating scan characterised by a broad and intense multi-peak melting endotherm in the range 30–180 °C, without a detectable stepwise specific heat increment that can be ascribed to the glass transition (Figure 3a,b, black curve). The low-T endotherm centered at 69 °C, typical of PVDF annealed at RT, has been ascribed to unstable secondary crystals [46] and disappears in the second heating scan (Figure 3c,d, black curve). After quenching the *T*_g_ of PVDF is just detectable at −45 °C. Conversely, PCADE (Figure 3a,b, green curve) is entirely amorphous, with a *T*_g_ = 58 °C characterised by a remarkable enthalpy relaxation peak due to physical ageing, that disappears in the second heating scan after quench (Figure 3c,d, green curve). As a general rule, in the case of immiscible blends there are two distinct glass transitions at temperatures matching those of the pure components, with magnitudes proportional to the corresponding component in the blend. In the first heating scans of both PVDF/PCADE blends (Figure 3a,b) no evident change of the baseline is detectable up to 30 °C. In the range 30–85 °C several phenomena apparently overlap: (i) the low-T melting endotherm of PVDF component, (ii) the *T*_g_ of the PCADE component and (iii) the relative relaxation peak due to physical ageing, whose intensity is higher for the blend richer in PCADE. In the range 140–180 °C the curves show the main endotherm melting of the PVDF crystal phase. The high tendency of the PVDF component to crystallise prevents the detection of its *T*_g_. In addition, the overlapping of thermal transitions of the pure components makes the interpretation of DSC curves in the first heating scan difficult. After quench (Figure 3c,d), the crystallinity of PVDF component is reduced and any physical ageing phenomenon is erased; both blends display a modest stepwise change of the baseline at −45 °C, corresponding to the *T*_g_ of pure PVDF, providing evidence of PVDF−PCADE phase separation, albeit in the range 30–85°C no remarkable differences can be detected. DMTA analysis was carried out to better shed light on the compatibility between PVDF and PCADE (Figure 3e). In the blends, the storage modulus shows two distinct and well-resolved decrements, detected at temperatures unaffected by blend composition and closely matching the pure components’ *T*_g_. Moreover, it is found that the magnitude of each decrement changes in proportion to the amount of the corresponding component in the blend, providing good evidence that PVDF and PCADE are immiscible in the fibres.

TGA analysis (Figure 3f) highlights that the two polymers thermally degrade in different temperature intervals: PVDF is stable up to 400 °C and shows two degradative steps; PCADE shows the onset of the first degradative step at about 180 °C and continuously loses weight up to complete degradation at 550 °C. In the blends, three main steps of degradation are detectable, the first one ascribable to PCADE and the others to PVDF. As the amount of PCADE in the fibres increases, the amount of material degraded in the first step of degradation increases as well. In particular, the nominal compositions of PVDF/PCADE at the weight ratios of 85/15 and 70/30 are confirmed by TGA analysis: 83/17 and 68/32 weight ratios, respectively.

XPS analysis was used to quantify the polymer composition at the fibre surface. The peak fitting elaboration was carried out on the sample C1s envelopes (Figure S6 and S7, Table S1). In the C1s envelope of PVDF/PCADE samples new peaks appear when compared with PVDF, in line with the different chemical composition of PVDF and PCADE. The relative abundance of new peaks was considered to calculate the surface composition. The experimental results differ significantly from the theoretical values calculated with the assumption that PCADE is homogeneously distributed along fibre cross-section (Table 1). While the lower F/C ratio could be due to the presence of hydrocarbon contamination, usually observed in XPS analysis of polymer systems, the higher oxygen content apparent in both O/C and F/O ratios could be explained by a preferential segregation of the PCADE onto the outer sample layers. The amount of PCADE present on the surface has been calculated from the F/O atomic ratio and from the correction from hydrocarbon contamination (Supplementary Materials). The results show that there is likely significant surface enrichment in PCADE.

**Figure 4:**
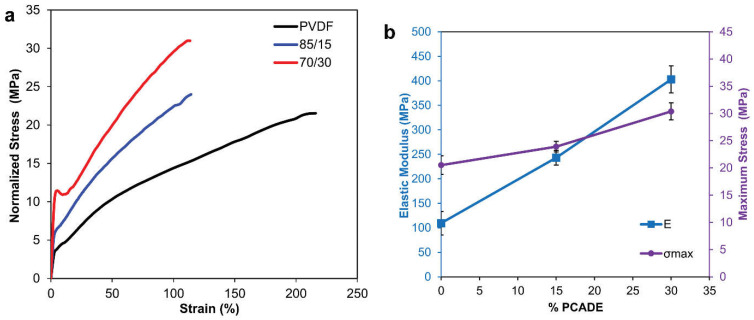
(**a**) Representative stress−strain curves of PVDF (black), PVDF/PCADE 85/15 (blue) and PVDF/PCADE 70/30 (red); (**b**) Elastic modulus and maximum stress as a function of PCADE weight content.

The mechanical behaviours of the three different electrospun mats are reported through stress−strain curves (Figure 4a and Table 2). It is pointed out that pure PCADE fibres were too fragile to be tested, as the mat could not be fixed to the clamps without being damaged. Stress−strain curves of the tested samples are characterised by a linear elastic region and a yield point followed by a plastic deformation. Both the elastic modulus and maximum stress at break grow linearly with increasing the percentage amount of the PCADE (Figure 4b). However, the absorbed strain energy is lower for the blends compared to the pristine PVDF nanofibres, due to the presence of the glassy phase of PCADE that reduces the maximum strain at break. Overall, the addition of PCADE confers a stiffer and stronger character to the fibres that, concomitantly, become less tough.

Overall, the different characterisation techniques highlighted that PCADE can be successfully electrospun with PVDF to produce nanometric fibres enriched at the surface with functionalisable epoxy groups in a one-step process. By changing blend composition, it is possible to tune the abundance of chemical functionalities at the surface and, at the same time, affect material bulk properties for high PCADE loading. Epoxy group can react with a variety of nucleophilic reagents (e.g., carboxylic acids and amines) and can be exploited in the framework of different applications, as described in the following sections.

### 3.2. Epoxide-Based Composites

Carbon Fibre Reinforced Polymers (CFRP) are structurally versatile materials employed in all fields where high specific strength is required [47]. Among them, the epoxide-based composites are widely used in load-bearing applications, e.g., automotive, aerospace, construction, because of their good mechanical properties, high specific strength and super adhesiveness as well as good heat and solvent resistance [48]. In the last decade, the specific features of electrospun fibres, namely high aspect ratio, uniform dispersion within the matrix and high surface area to volume ratio, makes them ideal candidates as second-phase fillers in CFRP laminates, proving to affect mechanical performances by improving, for instance, delamination resistance [49,50,51]. Different polymeric nanofibres, such as nylon 6,6 [52,53,54], and PVDF [55], have been successfully employed with a global effect of increasing mode I strain energy release rate (G_I_) and mode II strain energy release rate (G_II_), albeit reported results can be extremely variable [49]. Due to the complexity of these three-phase systems, a universal agreement on the reasons for changes in mechanical properties has still to be reached, but there seems to be a certain consensus about some factors [56]. In particular, (i) poor adhesion between fillers and matrix creates voids in the material, imposing a tortuous crack propagation [57,58], (ii) chemical interactions between filler and matrix cause substantial changes in the properties of the interfacial region [59,60,61,62] and (iii) the filler can slow down crack propagation by pinning and deflecting mechanisms, according to its size and tenacity [63,64,65].

**Figure 5:**
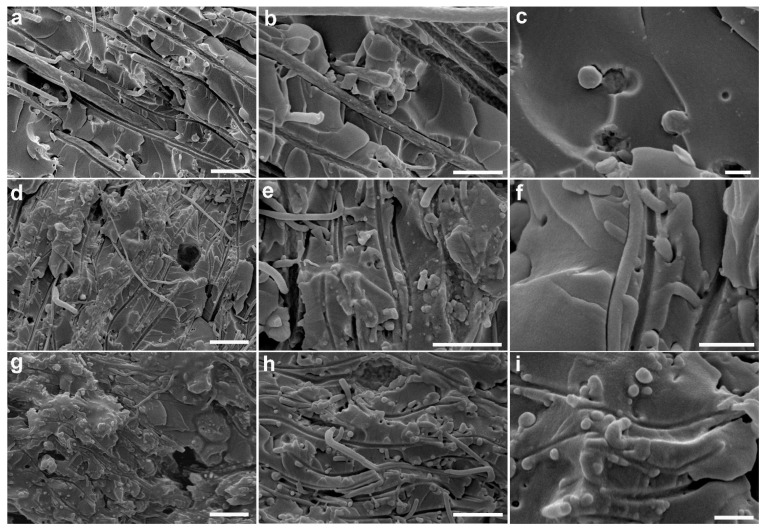
SEM images of electrospun fibres/epoxy matrix composites (cross-sections obtained by brittle fracturing in liquid nitrogen) after curing: (**a**–**c**) PVDF, (**d**–**f**) PVDF/PCADE 85/15 and (**g**–**i**) PVDF/PCADE 70/30. Scale bar = 10 μm (**a**,**d**,**g**); 5 μm (**b**,**e**,**h**); 2 μm (**c**,**f**,**i**).

Undoubtedly, the use of nanofibres determines a dramatic increase of the matrix–filler interface, hence good fibre–matrix interaction is crucial for achieving an efficient load transfer from polymer matrix to nanofibre fillers [66,67,68]. In this context, the epoxy-rich surface of PVDF/PCADE fibres is expected to provide a good interfacial bonding with an epoxy resin, promoting crosslinking across the interface by reacting with the amine groups of the hardener. SEM images of the epoxy resin filled with the different types of fibre are reported in Figure 5, as cross-sections obtained by brittle fracture in liquid nitrogen. As expected, the adhesion between the nanofibres and the epoxy matrix increases with the amount of PCADE in the fibres, this is evident by observing SEM images acquired at higher magnification.

**Figure 6:**
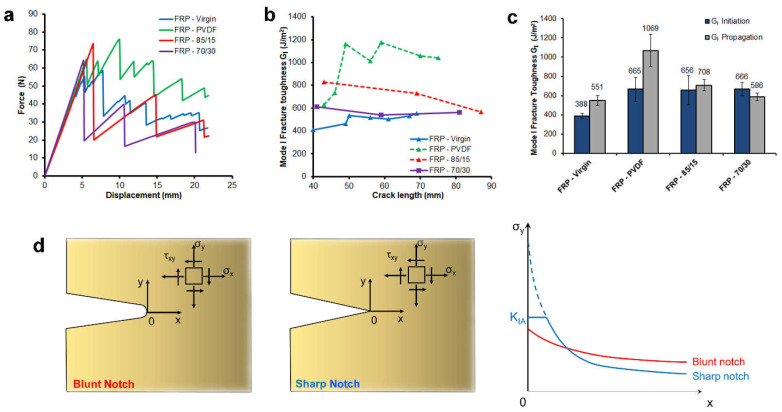
Delamination test results on virgin laminates and on laminates interleaved with PVDF, 85/15 and 70/30 blends: (**a**) representative force vs. displacement curves recorded during double cantilever beam (DCB) tests, (**b**) representative mode-I fracture toughness G_I_ vs. crack length (R-curve) and (**c**) graph bar comparison of the fracture toughness at crack initiation and propagation (average between 50 and 70 mm crack length). (**d**) Geometrical effect of the notch shape (blunt vs. sharp) on the stress distribution at the crack tip.

The mode I fracture toughness of the nano-interleaved composite laminates was evaluated by the DCB test. In the graph of force vs. displacement (Figure 6a) representative curves are shown, while in the graph of the corresponding mode I strain energy release rate (evaluated according to Equation (2)) vs. crack length, R-curves are reported (Figure 6b). Fracture toughness at crack initiation and propagation for each of the laminates are summarised in a bar graph (Figure 6c). The curves of force vs. displacement of the nano-interleaved PVDF/PCADE laminates show a fragile behaviour, with only a few sudden high force drops, while for the PVDF nano-interleaved laminates, the force after crack initiation is more stable. The fracture toughness at crack initiation is enhanced up to 70% by nano-interleaving, independently of polymer type. At crack propagation, pure PVDF fibres improve the fracture toughness of 94%, while for the 85/15 and 70/30 blends it is enhanced by only 29% and 6%, respectively.

The different R-curve trends between the laminates with PVDF and PVDF/PCADE could be explained by considering the effect of the crack tip shape (Figure 6d). At crack initiation, the notch artificially induced by a Teflon sheet is blunt and therefore crack initiation is less sensitive to the material toughness. Differently, at crack propagation, the crack tip is sharp and, for a purely elastic material, the stress in the proximity could rise theoretically at infinite, leading to breakage. A tough material, deforming plastically, can reduce the stress gradient at the crack tip hindering its propagation [69]. With this assumption, the effect of the different nanofibres on nano-interleaved composite mechanical properties can be explained in terms of fibre toughness—PVDF/PCADE blends, having a lower toughness compared to PVDF, (as evidenced by the tensile tests), can only increase the fracture toughness of the hosting laminate at crack initiation but slightly at propagation, when the crack becomes sharper. The result is that using PVDF/PCADE fibres it is possible to improve the fracture toughness of the composite laminate at damage initiation and, at the same time, the good adhesion with the matrix generates zones where the crack easily propagates upon the formation of sharp crack tips. Such a behaviour could be applied in the construction of the sacrificial part of safety devices, where a complete material failure is required once a specific force threshold is reached.

### 3.3. Enzyme Immobilisation

Enzyme membrane reactors (EMRs) are becoming increasingly important for application in bioconversion processes in food processing, pharmaceutics and biorefinery industries and in wastewater treatment. The major advantage of the EMRs is that they can achieve simultaneous enzymatic reaction and separation to reduce the production costs. Among all the different developed configurations of EMRs, immobilising enzymes directly in/on the membrane is a particularly important development of EMRs, as these systems usually display higher stability and reusability [70]. A series of immobilisation methods has been proposed to fix the enzymes on the membranes. The covalent immobilisation method usually results in stable enzymatic activities and good recycling reactors. The principle of the covalent method is to covalently link the enzymes to the membranes using functional groups that can be present on the membrane or be added by pretreating the membrane with functionalising reagents. The latter is the most common approach given the few naturally existing functional groups that can be directly used for covalent binding in most membranes [70]. In the present work, we have developed a covalent immobilisation strategy exploiting the epoxy groups introduced into PVDF/PCADE 70/30 electrospun fibres. Electrospun membranes have been proposed as suitable materials for enzyme immobilisation because of easy handling and recovery and high surface-to-volume ratios [71,72,73].

**Figure 7:**
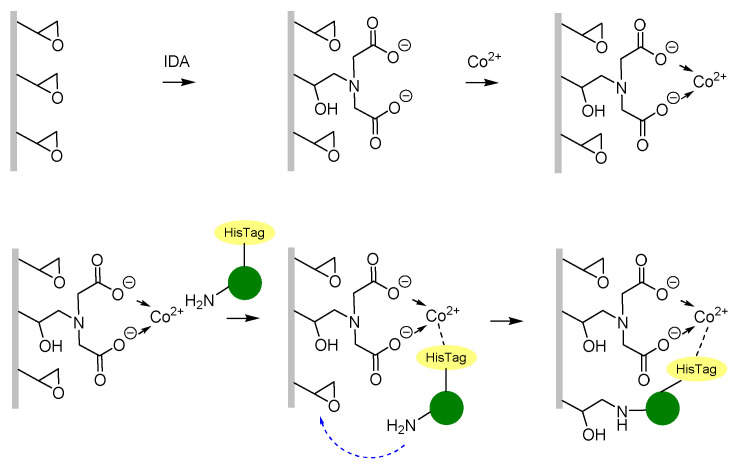
Covalent immobilisation of HeWT on epoxy membranes partially derivatised with metal by two step procedure: (1) affinity binding of his-tagged enzyme molecules to Co^2+^ groups, and (2) reaction of adjacent amine groups from lysine residues react with nearby epoxy groups.

We have applied an affinity-driven covalent immobilisation methodology to immobilise ω-transaminase enzyme from *Halomonas elongata* on these membranes to develop an EMR with potential use in heterogeneous biocatalysis. Firstly, epoxy groups at the fibre surface were chemically modified by iminodiacetic acid (IDA). This procedure did not affect fibre morphology (Figure S8) and enabled derivatisation of almost half of the epoxy group at the surface when carried out for 102 h (Supplementary Materials). The enzyme immobilisation strategy relies on a two-step process—the his-tagged enzyme molecules are driven by affinity towards Co^2+^ groups, enabling the solvent-exposed amine groups from lysine residues to react with nearby epoxy groups of the membrane (Figure 7) [35]. Two membranes were prepared targeting different degrees of metal-derivatisation by controlling the time of incubation with IDA, 2 h and 102 h, respectively, resulting in Co^2+^-2hIDA- and Co^2+^-102hIDA membranes after the metal coordination step. After the chemical treatments the electrospun membrane did not lose its integrity and apparently maintained its original mechanical properties and good handling. Calculated immobilisation parameters of HeWT on both membranes are shown in Table 3. Immobilisation of HeWT on Co^2+^-102hIDA membrane was successfully achieved showing a recovered activity value of 11.8 U g_membrane_^−1^, which is 43.6% of the immobilised activity. The same immobilisation strategy was previously applied to immobilise HeWT on Sepabeads^®®^ EC-EP/S, a polymetracrylate-based porous beads support, yielding 30–40% recovered activity [74,75]. However, the immobilisation yield obtained for the electrospun fibres was lower than the previously obtained immobilisation yield for polymetracrylate-based porous beads supports. This might be due to a limitation in the loading capacity of the membrane, which may display a lower epoxy group density, with respect to the important amount of enzyme offered (10 mg_HeWT_ g_membrane_^−1^). With respect to the immobilised enzyme only, 43% of the activity was recovered, meaning that a similar distortion of the structure is expected upon immobilization on the elctrospun fibres compared to the polymetracrylate-based porous beads supports. A lower degree of metal derivatisation is expected in the membrane treated with IDA for 2 h. Consequently, the immobilisation yield was lower in the Co^2+^-2hIDA- compared to the one obtained in the Co^2+^-102hIDA membrane. The immobilisation of HeWT on both membranes is covalent (i.e., amine groups from lysine residues and other reactive groups of the enzyme have reacted with epoxy groups of the membrane). This was shown by the fact that after incubating the imm-HeWT membranes for 1 h at 90 °C in liquid solution, no enzyme was observed in the liquid by SDS-page electrophoresis (data not shown), meaning that no enzyme subunit was detached from the membrane, and indicating that HeWT subunits were covalently bound to the membrane. Therefore, the method developed in this work for enzyme immobilisation on membranes has huge potential in the field of EMR and moves forward the use of this type of reactor in heterogeneous biocatalysis and other applications.

## 4. Conclusions

A novel bio-based polycarvone acrylate di-epoxide (PCADE) derived from terpene biomass has been employed as an additive in the electrospinning process of poly(vinylidene fluoride) (PVDF) to endow the fibres with functionalisable epoxy groups at their surface. Different amounts of PCADE have been incorporated in PVDF fibres, namely 15 and 30 % by weight, and fibre bulk composition was verified by TGA analysis. DSC and DMTA, taken together, enabled the thermal transitions of PVDF to be distinguished from the glass transition of PCADE, thus demonstrating that the two polymers are immiscible. Notably, XPS measured a surface composition PVDF/PCADE of 76/33 and 47/53 by weight, for the blend 85/15 and 70/30, respectively, thus showing that PCADE preferentially segregates at fibre surface during electrospinning. The proposed one-step approach makes use of a bio-based additive to confer new functionalities to electrospun fibres and it is potentially applicable to other types of polymeric systems to achieve ready-to-use surface-functionalisable fibres. The potentiality of the approach has been here tested in two different applications, by using PVDF/PCADE fibres as interleaves in CFRP and to develop membranes for heterogeneous enzyme catalysis. In the first case the presence of PCADE improves the adhesion between the electrospun fibres and the epoxy matrix, thanks to chemical crosslinks established at the interface. Mechanical properties of CFRP interleaved with PVDF/PCADE were found to be modulated by blend composition. In the second case ω-transaminase enzyme from *Halomonas elongata* was successfully covalently immobilised on the surface of the PCADE-loaded fibres opening opportunities in heterogeneous biocatalysis.

## Figures and Tables

**Table 1:** Surface composition of PVDF/PCADE blends by XPS analysis.

Electrospun Samples	Total PVDF/PCADE ^1^ (wt/wt)		F/C	O/C	F/O	Surface PVDF/PCADE ^2^ (wt/wt)
PVDF/PCADE 85/15	85/15	Theoretical ^3^	0.77	0.071	10.9	67/33
		Experimental ^4^	0.55	0.14	3.90	
PVDF/PCADE 70/30	70/30	Theoretical ^3^	0.58	0.13	4.5	47/53
		Experimental ^4^	0.32	0.19	1.7	

^1^ Nominal weight composition. ^2^ Surface composition by Equation S1. ^3^ Theoretical atomic ratios, calculated according to the nominal composition and repeat unit formulas, by assuming that PCADE distribution is homogeneous along fibre cross-section. ^4^ Experimental atomic ratios by XPS, after correction for contamination.

**Table 2:** Elastic modulus (E), maximum stress (σ_max_), maximum deformation (ε_max_) and absorbed strain energy (U) of the electrospun samples determined by stress−strain tests.

Electrospun Sample	E(MPa)	σ_max_ (MPa)	ε_max_ (%)	U (J/cm^3^)
PVDF	109 ± 24	20.5 ± 1.7	238 ± 18	32.0 ± 4.4
PVDF/PCADE 85/15	243 ± 15	23.9 ± 1	117 ± 8	19.0 ± 1.5
PVDF/PCADE 70/30	403 ± 28	30.4 ± 1.6	117 ± 11	23.9 ± 2.9

**Table 3:** Immobilisation of HeWT on metal-derivatised membranes, Co^2+^-2hIDA- and Co^2+^-102hIDA membranes.

Immobilisation Parameter	Co^2+^-2hIDA Membrane	Co^2+^-102hIDA Membrane
Offered enzyme (mg g_membrane_^−1^)	10	10
Immobilised activity (U g_membrane_^−1^) ^1^	10.7	27.2
Immobilisation yield(%) ^1^	24.4	61.9
Recovered activity (U g_membrane_^−1^) ^1^	2.9	11.8
Recovered activity (%)^1^	26.7	43.6

^1^ Immobilised activity (U g_membrane_^−1^), immobilisation yield (%), recovered activity (U g_membrane_^−1^) and recovered activity (%) were calculated as described in Materials and Methods.

## Supplementary Materials

The following are available online at https://www.mdpi.com/article/10.3390/polym13111804/s1, Figure S1, S2: chemical structures, Figure S3: curing process; Figure S4: electrospinning of pure PCADE, Figure S5: fibre diameter distributions; Figures S6 and S7: XPS analysis; Table S1: XPS data; Figure S8: SEM of IDA treated membrane.
