# Supplementary material for: Functionalisable Epoxy-rich Electrospun Fibres Based on Renewable Terpene for Multi-Purpose Applications
Source: Polymers (Basel). 2021 May 30;13(11):1804. doi: 10.3390/polym13111804 (PMC8198691; doi:10.3390/polym13111804)
Supplement: Supplementary file 1 [file polymers-13-01804-s001.zip › polymers-1236619-supplementary.pdf]

Supporting Information

Functionalisable Epoxy-rich Electrospun Fibres Based on Renewable Terpene for multi-purpose applications

Ulisse Montanari[a,b], Davide Cocchi[c,d], Tommaso Maria Brugo[c,d], Antonino Pollicino[e], Vincenzo Taresco[a], Maria Romero Fernandez[a], Jonathan C. Moore[a], Domenico Sagnelli[a], Francesca Paradisi[a,f], Andrea Zucchelli[c,d], Steven M. Howdle[a], Chiara Gualandi[b,d]

[a]School of Chemistry, University Park University of Nottingham, NG7 2RD, UK

[b]Department of Chemistry "Giacomo Ciamician" and INSTM UdR of Bologna, University of Bologna, Via Selmi, 2, 40126 Bologna, Italy

[c]Department of Industrial Engineering (DIN), Università di Bologna, Viale del Risorgimento 2, 40123 Bologna, Italy

[d]Interdepartmental Center for Industrial Research on Advanced Applications in Mechanical Engineering and Materials Technology, CIRI-MAM, University of Bologna, Viale Risorgimento, 2, 40136 Bologna, Italy

[e] D.I.C.A.R., Dipartimento di Ingegneria Civile e Architettura University of Catania, Viale Andrea Doria 6, 95125, Catania, Italy

[f]Department of Chemistry and Biochemistry University of Bern, Freiestrasse 3, Bern, Switzerland

1. Synthesis of poly(carvone acrylate di-epoxide)

3-Chloroperoxybenzoic Acid (*m*-CPBA, 70-75%), 2,2′-azobis(2-methylpropionitrile) (AIBN, 98%) dodecyl mercaptan (DDM, 98%), dichloromethane (DCM), deuterated chloroform ($CDCl_3$), tetrahydrofuran (THF), magnesium sulphate ($MgSO_4$), sodium sulfite (Na_2SO_3), sodium carbonate (Na_2CO_3) and sodium bicarbonate ($NaHCO_3$) were purchased from Sigma Aldrich and used without further purification.

Carvone acrylate (CA, Figure S1, 1) was synthesised as previously reported [1]. Epoxidation to synthetize carvone acrylate di-epoxide (CADE, Figure S1, 2) was then carried out. In a typical synthesis 200 mg of carvone acrylate (0.97 mmol) were dissolved in a suspension of 95 mg $NaHCO_3$ (1.1 mmol) in 10 mL DCM. 225 mg of m-CPBA (0.98 mmol) were dissolved in 5 mL

DCM and added dropwise to the suspension. After 6 h, 225 mg of m-CPBA (0.98 mmol), dissolved in 5 mL DCM, were added to the suspension and the reaction mixture was left overnight at room temperature (RT). The reaction was quenched with a solution of 150 mg Na_2SO_3 (1.2 mmol) in 15 mL H_2O. The organic layer was separated, washed three times with a 5% solution of Na_2CO_3, dried over $MgSO_4$ and evaporated under vacuum.

The desired CADE was obtained in 91% yield as a mixture of four diastereomers (Figure S2). The mixture was characterised by NMR: ^1H NMR (400 MHz, $CDCl_3$). δ 6.45 (1H) 6.13 (1H) 5.89 (1H) 5.28-5.08 (1H) 2.53 (2H) 1.31 (3H) 1.25 (3H) 2.25-1.05 (5H). J coupling and multiplet patterns unassigned due to overlapping of 4 diastereomeric protons per peak. ^{13}C NMR (400 MHz, $CDCl_3$): δ 165.9, 131.5, 128.3, 73.9, 60.6, 58.4, 58.3, 53.0, 39.1, 27.2, 25.4, 19.0, 17.9. The reported values are the average chemical shift of each cluster of four peaks [2].

Poly(carvone acrylate di-epoxide) (PCADE; Figure S1, 3) was synthetized by free radical polymerization. High molecular weight PCADE (M_n = 32000 g mol^{-1}, Đ = 2.1) was obtained by dissolving 1 g of CADE in 0,25 ml of a 4:1 THF/ tert-butyl alcohol mixture with 5 mg of AIBN. Low molecular weight PCADE (M_n = 2800 g mol^{-1}, Đ = 1.5) was synthetized by dissolving 1 g of CADE in 2 mL of a 2:1 THF:propanol mixture with 5 mg of AIBN and 50 mg of dodecane thiol as transfer agent. The reaction mixture was degassed for 30 min before heating at 65 °C for 18 h. The polymer was purified by precipitation in excess cold methanol.

PCADE: ^1H NMR (400 MHz, $CDCl_3$). δ 4.80-4.60 (1H, m) 2.70-2.40 (2H, m) 5.89 (1H) 2.10-0.70 (15H, m).

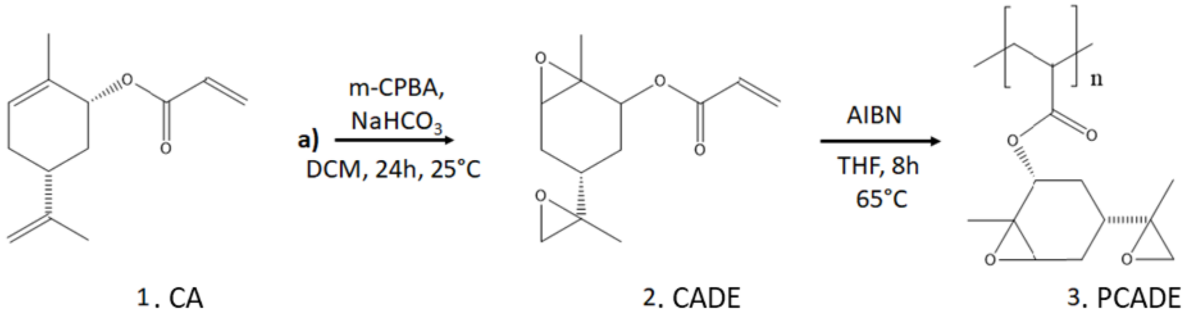

Figure S1. a) Carvone acrylate (CA) epoxidation for the synthesis of CADE and b) polymerisation for the synthesis of PCADE.

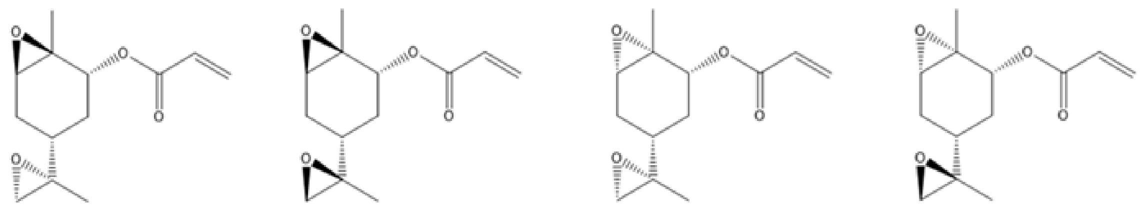

Figure S2. Chemical structures of the four diasteroisomers obtained as product in the synthesis of Carvone acrylate di-epoxide.

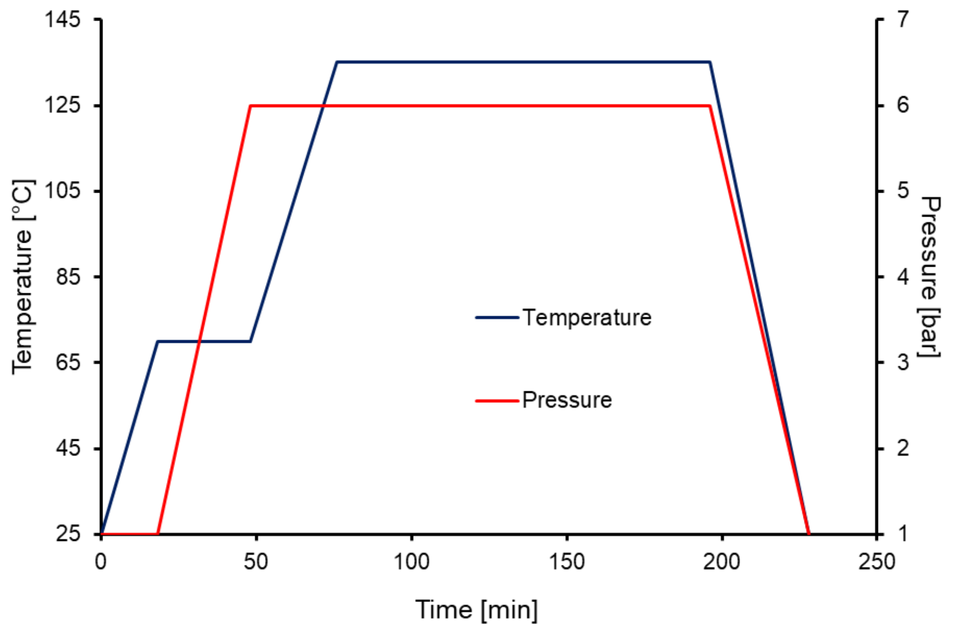

Figure S3. Curing process of laminate composites.

3. Electrospinning of PCADE

High molecular weight PCADE was electrospun from a solution in THF:DMF=2:1 (v/v) at different concentrations. As expected, drastic morphological changes were observed when polymer concentration increases: at low concentration of 10 % w/v beaded discontinuous fibres were obtained (Figure S4a and b), while by increasing polymer concentration to 20 % w/v beads disappeared along the fibres that, concomitantly, experienced an evident increase of their diameters (Figure S4c and d). However, bead-free PCADE fibres are discontinuous as a consequence of the fragile nature of the polymer.

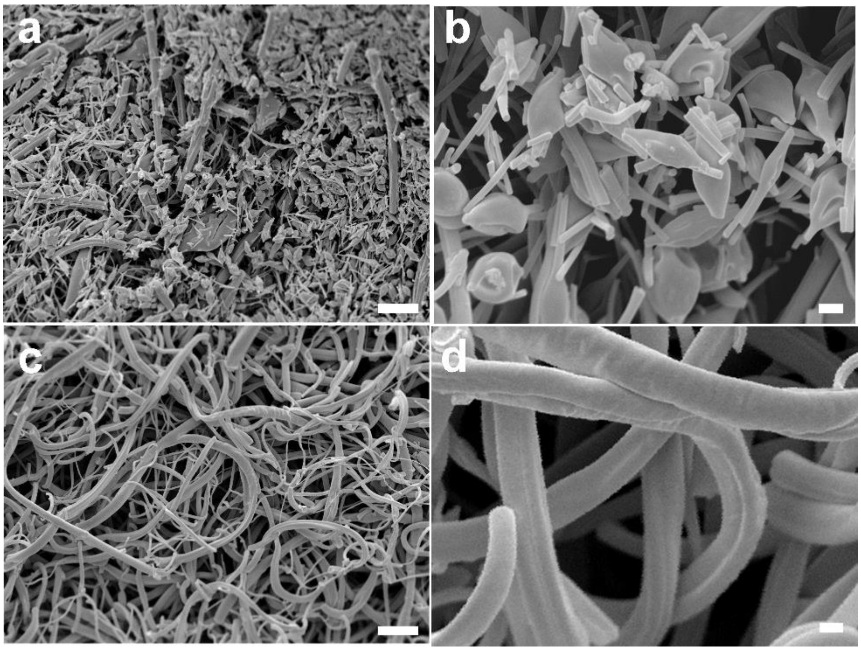

Figure S4. SEM images of high-molecular weight PCADE electrospun from a mixed solvent of THF:DMF=2:1 at a concentration of 10% w/v (a and b) and of 20% w/v (c and d). Scale bars: 20 μm (a and c); 2 μm (b and d).

4. Fibre diameter distribution

The fibre average diameter has been determined by SEM analysis, measuring at least 150 fibres for each sample. Results provided in Figure S5 show that fibre diameter distributions are statistically different ($p < 0.001$).

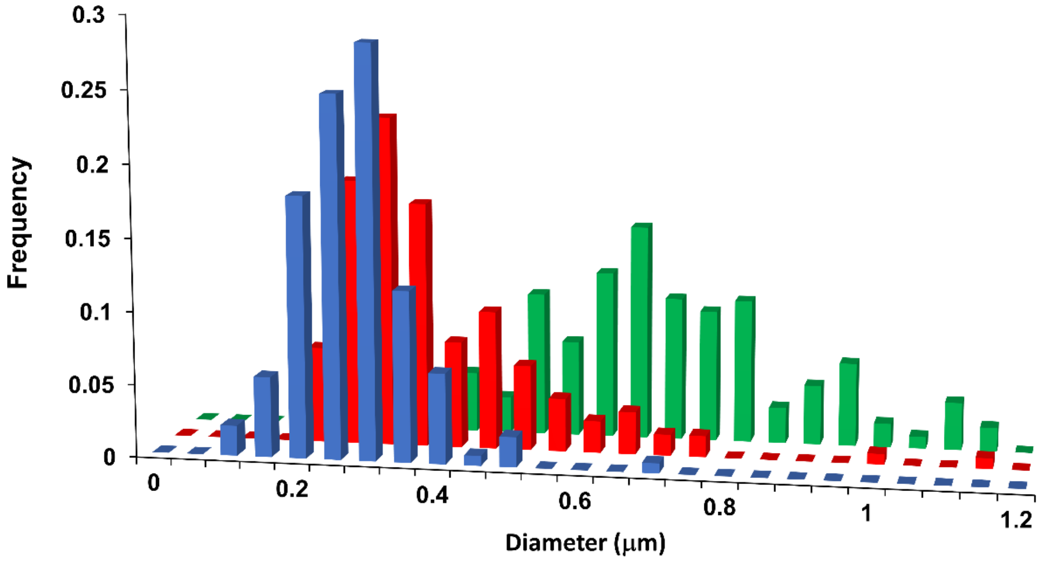

Figure S5. Fibre diameter distributions of PVDF (green), PVDF/PCADE 85/15 (red) and PVDF/PCADE 70/30 (blue).

5. X-Ray Photoelectron Spectroscopy

The PVDF/PCADE blend widescans (Figure S6) shows the presence on the surface of carbon (~285 eV), oxygen (~532 eV) and fluorine (~688 eV).

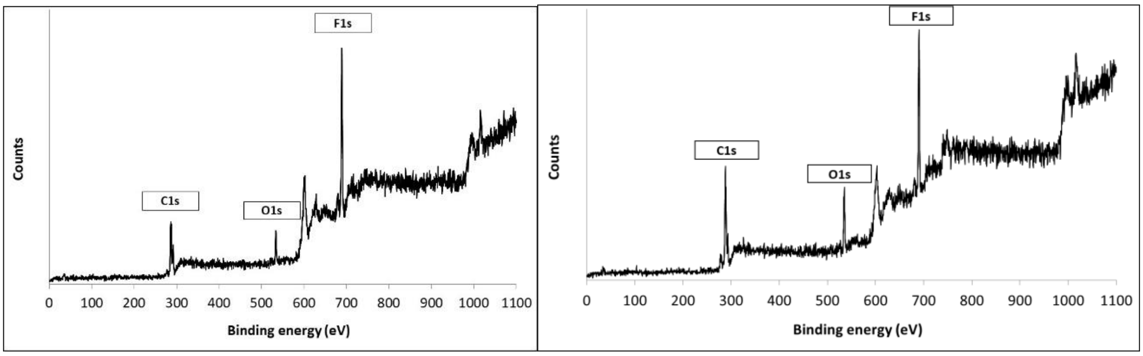

Figure S6. Widescans of a) PVDF/PCADE 85/15 and b) PVDF/PCADE 70/30.

From the feeding ratio and repeat unit formulas ($C_2H_2F_2$ / $C_{13}H_{18}O_4$, respectively) the expected atomic ratios have been calculated and reported in Table 1 of the Manuscript.

To calculate the PCADE abundance from the F/O the Equation S1 has been used:

$$\frac{F}{O} = \frac{2 \cdot \frac{100 - PCADE\,wt\%}{MW_{PVDF}}}{4 \cdot \frac{PCADE\,wt\%}{MW_{PCADE}}} \qquad [S1]$$

Where MW_{PVDF} is the molecular weight of the PVDF repeat unit, and MW_{PCADE} is the molecular weight of the PCADE repeat unit.

The correction of the carbon abundance from hydrocarbon contamination has been carried out through a curve fitting elaboration of the C_{1s} envelope (Figure S7). From the structure of the repeat units seven chemical states can be identified and their position and relative abundance can be predicted:

- C1 (285 eV) deriving from the 6 carbon atoms, present in PCADE repeat unit, bound to other carbon or hydrogen atoms (C-C or C-H);
- C2 (285.4 eV) deriving from the carbon atom in α with respect to carboxylic function of PCADE repeat unit;
- C3 (286.5 eV) deriving from the carbon atom, present in PCADE repeat unit, bonded with single bond to the ester oxygen atom;
- C4 (286.6 eV) deriving from the carbon atom, present in the repeat unit of PVDF, bonded to hydrogen;

- C5 (287 eV) deriving from the 4 carbon atoms, present in PCADE repeat unit, of the epoxy functions;
- C6 (289 eV) deriving from the carboxylic carbon atom, present in PCADE repeat unit;
- C7 (290.9 eV) deriving from the carbon atom, present in the repeat unit of PVDF, bonded to fluorine.

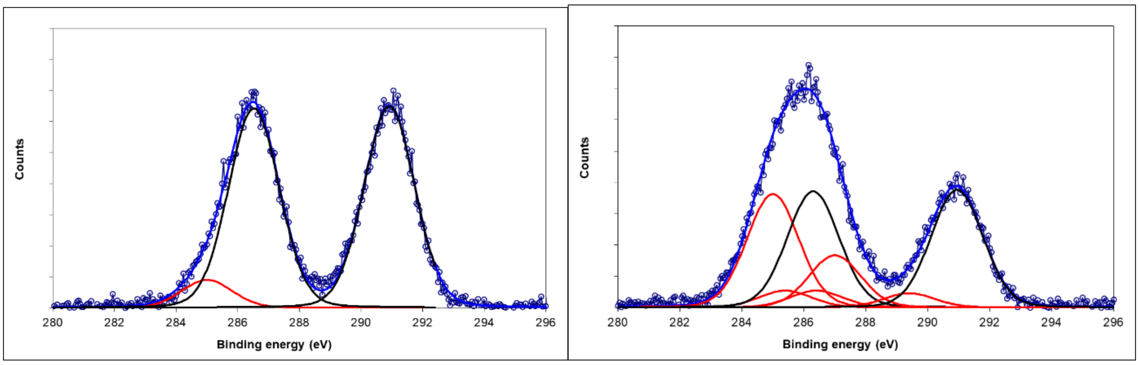

Figure S7. Peak fitting of the C1s envelope of sample PVDF (right) and PVDF/PCADE 85:15 (left).

The relative abundance of the carbon function as obtained from the curve fitting of the C_{1s} envelope are reported in Table S1.

Table S1. Relative abundance of the carbon function as obtained from the curve fitting of the C_{1s} envelope.

Sample		C1	C2	C3	C4	C5	C6	C7
PVDF/PCADE 85/15	Obtained from curve fitting	25%	3.8%	3.8%	26%	12%	3.0%	26%
	Calculated from F/O ratio	21%	3.5%	3.5%	27%	14%	3.5%	27%
PVDF/PCADE 70/30	Obtained from curve fitting	42%	4.0%	4.0%	17%	13%	4.0%	17%
	Calculated from F/O ratio	30%	5.0%	5.0%	17%	20%	5.0%	17%

From these results a hydrocarbon contamination of 4% and 9% can be calculated for PVDF/PCADE 85/15 and 70/30, respectively, and then corrected atomic ratios obtained by subtraction of this contamination from the C_{1s} contribution.

The surface analysis of PVDF/PCADE 70:30 IDA treated sample (120 h) confirmed the success of the reaction. In fact, while the surface analysis of IDA treated PVDF fibers did not show any nitrogen, the surface of PVDF/PCADE 70:30 IDA treated sample, corrected from the hydrocarbon contamination, has the following atomic percent composition C 55,6%, F 22,7%, O 20.6%, N 1,2%, while the expected one for a complete IDE/epoxy reaction is C 55,4%, F 22,7%, O 18.8%, N 3,1%, meaning that almost half of the epoxy groups were functionalized.

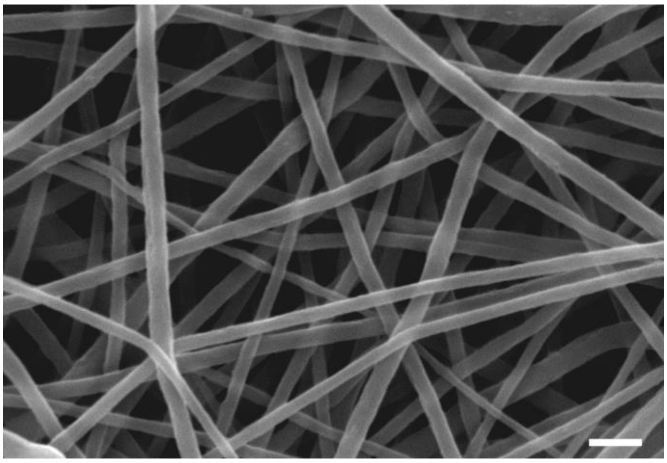

Figure S8. SEM image of PVDF/PCADE 70/30 chemically modified by a solution of iminodiacetic acid (IDA) for 102 h. Scale bar = 1 μm.

References

[1] M.F. Sainz, J.A. Souto, D. Regentova, M.K.G. Johansson, S.T. Timhagen, D.J. Irvine, P. Buijsen, C.E. Koning, R.A. Stockman, S.M. Howdle, A facile and green route to terpene derived acrylate and methacrylate monomers and simple free radical polymerisation to yield new renewable polymers and coatings, Polym. Chem. 7 (2016) 2882–2887. https://doi.org/10.1039/C6PY00357E.

[2] U. Montanari, V. Taresco, A. Liguori, C. Gualandi, S.M. Howdle, Synthesis of novel carvone (meth)acrylate monomers for the production of hydrophilic polymers with high terpene content, Polym. Int. 70 (2021) 499–505. https://doi.org/https://doi.org/10.1002/pi.6096.
